# Identification and Distribution of Novel Cressdnaviruses and Circular Molecules in Four Penguin Species in South Georgia and the Antarctic Peninsula

**DOI:** 10.3390/v12091029

**Published:** 2020-09-16

**Authors:** Hila Levy, Rafaela S. Fontenele, Ciara Harding, Crystal Suazo, Simona Kraberger, Kara Schmidlin, Anni Djurhuus, Caitlin E. Black, Tom Hart, Adrian L. Smith, Arvind Varsani

**Affiliations:** 1 Department of Zoology, University of Oxford, South Parks Road, Oxford OX1 3SZ, UK; hila.levy@zoo.ox.ac.uk (H.L.); tom.hart@zoo.ox.ac.uk (T.H.); 2 The Biodesign Center for Fundamental and Applied Microbiomics, Center for Evolution and Medicine, School of Life Sciences, Arizona State University, Tempe, AZ 85287-5001, USA.; rafasfontenele@asu.edu (R.S.F.); chardin5@asu.edu (C.H.); csuazo@asu.edu (C.S.); simona.kraberger@asu.edu (S.K.); Kara.Schmidlin@asu.edu (K.S.); 3 Faculty of Science and Technology, University of the Faroe Islands, Vestarabryggja 15, FO-100 Tórshavn, Faroe Islands; anni.djurhuus@gmail.com; 4 Department of Evolutionary Biology and Environmental Studies, University of Zurich, Winterthurerstrasse 190, 8057 Zurich, Switzerland; caitlin.black@uzh.ch; 5 Structural Biology Research Unit, Department of Integrative Biomedical Sciences, University of Cape Town, Observatory, Cape Town 7701, South Africa

**Keywords:** *Cressdnaviricota*, circular molecules, penguins, viruses, Antarctica, *Pygoscelis antarcticus*, *Pygoscelis adeliae*, *Pygoscelis papua*

## Abstract

There is growing interest in uncovering the viral diversity present in wild animal species. The remote Antarctic region is home to a wealth of uncovered microbial diversity, some of which is associated with its megafauna, including penguin species, the dominant avian biota. Penguins interface with a number of other biota in their roles as marine mesopredators and several species overlap in their ranges and habitats. To characterize the circular single-stranded viruses related to those in the phylum *Cressdnaviricota* from these environmental sentinel species, cloacal swabs (*n* = 95) were obtained from King Penguins in South Georgia, and congeneric Adélie Penguins, Chinstrap Penguins, and Gentoo Penguins across the South Shetland Islands and Antarctic Peninsula. Using a combination of high-throughput sequencing, abutting primers-based PCR recovery of circular genomic elements, cloning, and Sanger sequencing, we detected 97 novel sequences comprising 40 ssDNA viral genomes and 57 viral-like circular molecules from 45 individual penguins. We present their detection patterns, with Chinstrap Penguins harboring the highest number of new sequences. The novel Antarctic viruses identified appear to be host-specific, while one circular molecule was shared between sympatric Chinstrap and Gentoo Penguins. We also report viral genotype sharing between three adult-chick pairs, one in each Pygoscelid species. Sequence similarity network approaches coupled with Maximum likelihood phylogenies of the clusters indicate the 40 novel viral genomes do not fall within any known viral families and likely fall within the recently established phylum *Cressdnaviricota* based on their replication-associated protein sequences. Similarly, 83 capsid protein sequences encoded by the viruses or viral-like circular molecules identified in this study do not cluster with any of those encoded by classified viral groups. Further research is warranted to expand knowledge of the Antarctic virome and would help elucidate the importance of viral-like molecules in vertebrate host evolution.

## 1. Introduction

Our knowledge of global viral diversity remains limited, with only a small fraction of viruses known to affect humans and wildlife having been properly described or studied. In the case of Antarctic associated viruses, only a speck of the proverbial iceberg of viral diversity has surfaced in modern research. The Antarctic Polar Front, the marine boundary between the Antarctic and sub-Antarctic waters formed in the Eocene, has played a part in the evolutionary divergence and high levels of endemism present in the Southern Ocean [1,2]. Penguins (Order: Sphenisciformes) are diving seabird mesopredators distributed across the islands and continental landmass of the Southern Ocean who form an important part of the avian biomass in the region.

Our understanding of diversity in the vertebrate hosts of the Antarctic region remains limited, with early research using serological techniques typically focusing on well-known avian and poultry pathogenic viruses associated with influenza, Newcastle Disease, and infectious bursal disease [3,4,5,6] or distemper viruses likely transferred from sled dogs to Antarctic pinnipeds [7,8]. A small number of symptomatic events linked to viruses, such as avian pox and “puffinosis-like” disease, have also been recorded in case reports [9,10,11]. Nonetheless, our knowledge of existing Antarctic vertebrate viruses beyond these reports remained constrained until the advent of high-throughput sequencing techniques, uncovering a growing number of viral genomes in the last decade, most recently reviewed by Smeele et al. [12] and complemented with the identification of RNA viruses in penguins and their ectoparasites [13].

Recent high-throughput sequencing metagenomic analysis of soil [14,15,16], lacustrine [17,18,19,20,21,22], marine [23,24,25], and ice-associated samples [26] in Antarctica have revealed robust viral profiles of genomes with novel characteristics. With an aim to uncover further viral diversity associated with penguins south of the Polar Front, we undertook a study to investigate the diversity of DNA viruses and viral-like circular molecules in four penguin species: The Adélie Penguin (*Pygoscelis adeliae*), Chinstrap Penguin (*P. antarcticus*), Gentoo Penguin (*P. papua*), and King Penguin (*Aptenodytes patagonicus*).

King Penguins are primarily sub-Antarctic seabirds with a circumpolar distribution on islands near the Polar Front. Adélie Penguins are considered true Antarctic, ice-loving birds, breeding along the entire Antarctic coast and southern islands of the Scotia Arc. Chinstrap Penguins, on the other hand, are restricted to South Georgia and the South Sandwich Islands, South Orkney Islands, South Shetland Islands, and Western Antarctic Peninsula. The widest-ranging penguin species is the Gentoo Penguin, with a circumpolar distribution that spans 46–66° S, breeding north and south of the Polar Front on Sub-Antarctic Islands and throughout the entire Scotia Arc and down the Western Antarctic Peninsula. Some of the four species, therefore, overlap in portions of their range and provide an opportunity to investigate circular single-stranded DNA viruses related to those in the phylum *Cressdnaviricota* [27] across sites and in sympatric breeders. We also evaluated whether viral sequences are shared between parent-chick pairs of Pygoscelid penguins.

## 2. Materials and Methods

### 2.1. Field Sampling

**Figure 1:**
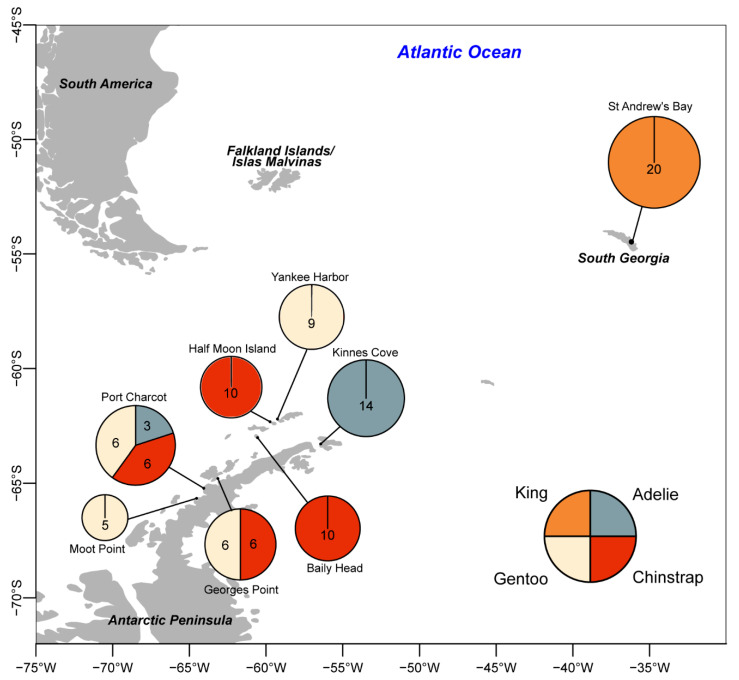
Sampling sites of the four-penguin species based on their breeding colonies in South Georgia and the Antarctic Peninsula. The numbers in the pie chart indicate the number of individual samples per species.

Between 22 December 2015 and 17 January 2016, a total of 95 cloacal swab samples were obtained as part of gastrointestinal microbial analysis from penguins. Since we had an archived sample set, we decided to determine the associated DNA viruses from the animals where none of the animals had any obvious signs of illness/disease at the 8 sites on Deception Island, the South Shetland Islands, South Georgia, and the Antarctic Peninsula (Supplementary Table S1, Supplementary Data 1). These consisted of 17 Adélie Penguins (*Pygoscelis adeliae:* 9 adults, 8 chicks), 32 Chinstrap Penguins (*P. antarcticus*: 21 adults, 11 chicks), 26 Gentoo Penguins (*P. papua*: 16 adults, 10 chicks), and 20 King Penguins (*Aptenodytes patagonicus*: 10 adults, 10 chicks) (Figure 1). Regular flocked swabs (Eswab™, Copan^®^, Murrieta, CA, USA) were used, stored in 1 mL of liquid Amies medium, which was stored frozen aside from transport with cold packs to the United Kingdom. Adult and juvenile King Penguins were sampled using a 2-person handling technique as they transited within the colony. Pygoscelid penguins were sampled in transit to or from the ocean, or where applicable, while on the nest in order to confirm chick-adult parentage, minimizing disturbance as much as possible and ensuring the parents returned to their chicks.

Sampling sites are shown in Figure 1, and details are found in Supplementary Table S1 and Supplementary Data 1. All sampling carried out in the Antarctic Treaty Area was performed in accordance with United Kingdom Home Office guidelines, under permits granted by the United Kingdom Foreign and Commonwealth Office (permits S3 04/2014 and S7 03/2014) and in South Georgia under permits granted by the Government of South Georgia and the South Sandwich Islands (RAP-2015-018), with ethical approval from the University of Oxford Animal Welfare and Ethical Review Board. All samples were transported in accordance with applicable export permits and United Kingdom Department for Environment, Food, and Rural Affairs import permits.

### 2.2. Viral Extraction and High-Throughput Sequencing Analysis

Viral DNA was extracted from 200 µL of the swab suspension after vortexing, using the High Pure Viral Nucleic Acid Kit (Roche Diagnostics, Indianapolis, IN, USA). Following viral DNA extraction, circular DNA was preferentially amplified by rolling circle amplification (RCA) using the TempliPhi™ 100 Amplification Kit (GE Healthcare, Chicago, IL, USA). An aliquot of the resulting RCA-amplified DNA (5 µL) was pooled based on animal species per site and used to generate Illumina sequencing libraries using the Nextera DNA Flex Library Prep Kit (Illumina Inc, San Diego, CA, USA). The libraries were sequenced on an Illumina 4000 sequencer (2 × 100 bp library) and the resulting paired-end raw reads were trimmed using Trimmomatic [28] with default settings. The trimmed reads were *de novo* assembled using metaSPAdes v 3.12.0 [29]. All assembled contigs >500 nts were analyzed against a viral RefSeq [30] protein database using BLASTx [31]. Circular contigs were determined based on terminal redundancy. A summary of the read counts and contigs > 500 nts per sample sequenced is provided in Supplementary Table S2. The raw reads have been deposited in SRA (SRX9081836–SRX9081846).

### 2.3. Recovery and Sequencing of Viral Genomes and Viral-Like Circular Elements

There were 28 cressdnavirus-like contigs identified, and these were used to design abutting primer pairs (Supplementary Table S3) for the recovery of complete viral genomes by PCR. The specific primers pairs, together with KAPA HiFi HotStart DNA Polymerase (Kapa Biosystems, Wilmington, MA, USA), were used to screen and amplify the virus genomes and viral-like elements from each sample, using the thermal cycling protocol recommended by the manufacturer with an annealing temperature of 60 °C and 0.5 µL of the RCA product. The resulting amplicons were resolved on a 0.7% agarose gel, excised, purified, and cloned into pJET1.2 plasmid (ThermoFisher, Waltham, MA, USA). The recombinant plasmids were Sanger-sequenced by primer walking at Macrogen Inc. (Seoul, Korea), and contigs assembled using Geneious Prime [32].

### 2.4. Bioinformatic Analyses of Recovered Viral Genomes and Viral-Like Elements

The open reading frames (ORFs) in the viral genomes and viral-like elements were identified using ORFfinder (https://www.ncbi.nlm.nih.gov/orffinder/) and annotated using Geneious Prime [32]. All pairwise identities (genome-wide and protein) were determined using SDTv1.2 [33].

A dataset of the replication-associated proteins (Rep) encoded by the members of the recently established phylum *Cressdnaviricota* [27], i.e., alphasatellites, circoviruses, geminiviruses, genomoviruses, nanoviruses, redondoviruses, and smacoviruses was assembled with sequences available in GenBank. This dataset was clustered with a 0.9 sequence identity cut-off using CD-HIT [34] and a representative from each cluster was used to build a Rep-CRESS dataset together with all the Rep sequences of unclassified CRESS DNA viruses and plasmids analyzed in Kazlauskas et al. [35]. The Rep-CRESS dataset, together with 54 Reps from this study, was used to generate a sequence similarity network (SSN) analysis using EST-EFI [36,37] with a minimum similarity score of 60. The SSN was visualized in an organic layout with Cytoscape V3.7.1 [38].

Similarly, a capsid protein (CP) dataset of the CPs encoded by circoviruses, geminiviruses, genomoviruses, nanoviruses, redondoviruses, and smacoviruses was assembled and clustered with a 0.9 sequence identity cut-off using CD-HIT [34]. Representative sequences from each cluster were assembled together with the RNA virus CP sequences (since they have similarities to some CRESS DNA virus CPs) of tombusviruses (*n* = 54; derived from RefSeq), albetoviruses (*n* = 3; derived from RefSeq), all CPs of unclassified CRESS DNA viruses, and 84 CPs from this study to build a CP-CRESS-albeto-tombus dataset. The CP-CRESS-albeto-tombus dataset was used to generate a sequence similarity network (SSN) analysis using EST-EFI [36,37] with a minimum similarity score of 10. The SSN was visualized in an organic layout with Cytoscape V3.7.1 [38].

Sequences within a network cluster that included sequences from this study were extracted and aligned using MAFFT [39,40]. The alignments were trimmed using TrimAl [41] with a gappy option, and the resulting alignments were used to determine the best amino acid substitution model using ProtTest [42] and infer Maximum likelihood (ML) phylogenetic trees with aLRT branch support. All trees were mid-point rooted, and branches with <0.8 aLRT support were collapsed using TreeGraph2 [43]. The trees were visualized using iTOL v4 [44].

Species-level sequence alignments of Antarctic viruses (AntVs) and Antarctic circular molecules (AntCMs) identified in this study were used to identify any evidence of recombination using RDP4 v.4.97 [45] with default settings. Only events that were detected by 3 or more recombination detection methods implemented in RDP v4.97 with *p*-values < 0.05 were accepted as credible.

## 3. Results and Discussion

### 3.1. Identification of Viruses and Viral-Like Molecules from Cloacal Swabs

We identified 28 cressdnavirus-like contigs >500 nts from the *de novo* assembled sequences from pooled samples per species per site that had similarity to viral sequences. In addition, we identified 24 bacteriophage-like contigs (8 inovirus-like, 11 microvirus-like, 1 mycovirus-like, podovirus-like, and 3 siphovirus-like). For the purpose of this study, we focused on the likely eukaryotic viruses that were most closely related to those in the phylum *Cressdnaviricota*. Using abutting primers designed from the *de novo* assembled contigs, we amplified, cloned, and Sanger-sequenced 40 viral genomes (with detectable Rep and CP-coding ORFs) and 57 viral-like elements. The 40 viral genomes (2322–2729 nts; GenBank accession numbers MT196222–MT196223, MT196247–MT196250, MT196252–MT196253, MT196261, MT196279, MT196289–MT196318) did not fall within any known viral families, and based on their Rep sequences, would be part of the expanding unclassified circular Rep-encoding single-stranded (CRESS) DNA viruses within the recently established phylum *Cressdnaviricota* [27]. The phylum *Cressdnaviricota* includes seven viral families (*Bacilladnaviridae*, *Circoviridae*, *Geminiviridae*, *Genomoviridae*, *Nanoviridae*, *Redondoviridae* and *Smacoviridae*), the Reps of the satellite nucleic acids in the family *Alphasatellitidae*, and a suite of viral groups that have been loosely labeled CRESS DNA viruses. Within these named viral families in the order *Cressdnaviricota*, only two Penguin circovirus genomes (family *Circoviridae*) had previously been recovered from this same sample set, found in a Chinstrap Penguin and an Adélie Penguin [46]. CRESS DNA viruses that cannot be classified into the eight families above have been identified primarily through viral metagenomic approaches from a variety of samples, including animal tissues, as highlighted in the recent report by Tisza et al. [47], fecal samples [48,49,50] and environmental samples including plant leaves [51], soil [52], wastewater [53,54,55], seawater [56], freshwater [57], and sea spray [58]. In Antarctica, CRESS DNA viruses have been identified from cryoconite samples from the dry valley [26], Adélie and Chinstrap penguin fecal and cloacal swab samples [46,59], lake samples [17], and a pond sample [22].

**Figure 2:**
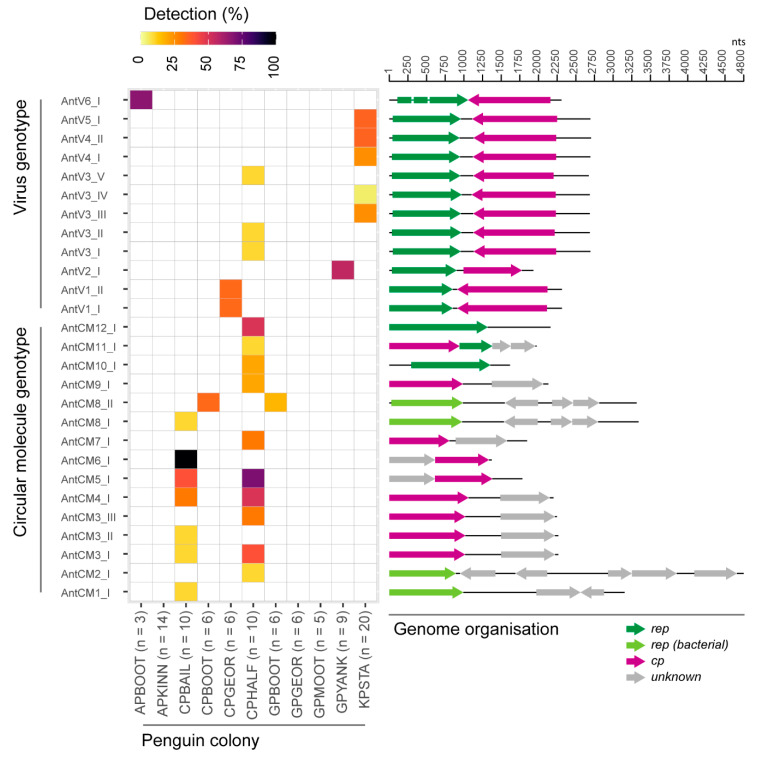
Heat map illustrating the dataset-wide detection scaled to sample size, of virus and circular molecule genotypes with the linearized genome organization shown on the right. The detection is based on the recovery of complete sequences by PCR, cloning, and Sanger sequencing. For the purpose of this study, the viruses and circular molecules have been classified as unique species based on a cutoff threshold of 80% genome-wide pairwise identity and genotypes based on 98% genome-wide pairwise identity. The penguin colony key is as follows: (first two letters) AP = Adélie Penguin, CP = Chinstrap Penguin, GP = Gentoo Penguin, and KP = King Penguin, followed by locations BOOT = Booth Island (Western Antarctic Peninsula), KINN = Kinnes Cove (Northern Antarctic Peninsula), BAIL = Baily Head (Deception Island), GEOR = Georges Point (Western Antarctic Peninsula), HALF = Half Moon Island (South Shetland Islands), MOOT = Moot Point (Western Antarctic Peninsula), YANK = Yankee Harbor (South Shetland Islands), and STA = St Andrews Bay (South Georgi).

Of 57 circular viral-like elements identified in this study, one viral-like element (1982 nts; MT196283) encodes a CP and a partial Rep (missing the N terminus, which is the endonuclease domain) and two additional unknown proteins, while eight (1243–2181 nts; MT196267–MT196271, MT196283, MT196286–MT196287) encode only a Rep. Six of the circular viral-like elements (2646–4800 nt; MT196226, MT196234-MT196236, MT196251, MT196288) encode Reps more similar to plasmid Reps. Finally, 43 viral-like elements (1759–2274 nts; MT196224–MT196225, MT196227–MT196233, MT196235–MT196254, MT19660, MT196262–MT196266, MT196272–MT196278, MT196280–MT196282, MT196284–MT196285) encode a CP and an unknown protein (Figure 2).

For the purpose of this study, we adopted an 80% genome-wide pairwise identity cutoff (similar to other CRESS DNA viruses) as a putative species demarcation cutoff and 98% identity for genotypes (Supplementary Data 2). Thus, 40 viral genomes represent 6 tentative species (labeled as Antarctic viruses, AntV 1–6) and 12 genotypes (Table 1). The circular molecules (labeled as Antarctic circular molecules, AntCM 1–12) can be split into 12 species and 15 genotypes (Table 1).

A preliminary BLASTn based analysis revealed that only AntCM3, -4, -5, -6, -7, -9, 10 and -12 have hits with >20% sequence coverage to viral-like sequences identified in sea water samples from Saanich Inlet (BC, Canada) [56], fish and abalone tissue [47]. The results of this are summarized in Table 2. JX904537 does not have annotated ORFs thus, is not included in the coding sequence analysis in this study.

### 3.2. Distribution of Viruses and Viral-Like Molecules across Sampling Sites and Penguin Species

Of 95 swab samples tested in this study, the 97 viral genomes and circular molecules were recovered from 45 individuals (2/17 Adélie Penguins, 24/32 Chinstrap Penguin, 6/26 Gentoo Penguins, and 13/20 King Penguins) with at least one AntV or AntCM (Table 1; Figure 2).

The 40 AntVs were recovered across the four penguin species (Table 1). From the 57 AntCMs recovered, 8 sequences that encode Reps were recovered only from Chinstrap Penguins while the other 49 sequences were recovered from Chinstrap Penguins and one Gentoo Penguin (Table 1; Figure 2).

AntCMs (12 species/15 genotypes) were found in 19/32 individual Chinstrap Penguins sampled and in a single Gentoo Penguin (Table 1; Figure 2). Seven AntCM genotypes were found in multiple individuals, with three genotypes shared across the two Chinstrap colonies sampled in the South Shetland Islands. AntCV6 genotype I was found in 100% (*n* = 10) of Chinstrap Penguins shared between Chinstrap Penguin parents and their chicks at Baily Head, Deception Island. One set of AntCM genotypes with a bacterial *rep* (AntCM8 genotype I and AntCM8 genotype II) at the edge of the species threshold (78.8–79.1% genome-wide identity) were found at two sites 290 km apart (Booth Island, Western Antarctic Peninsula and Deception Island, South Shetland Islands). AntCM8 genotype II was unique in this study as being the only genotype found in two distinct host species: An adult Gentoo Penguin and two adult Chinstrap Penguins nesting at the same colony on Booth Island, with >99.9% shared genome-wide identity. One of these adult Chinstrap Penguins was recently found also to carry a penguin circovirus [46]. Three species (each as a single genotype) of Antarctic circular molecules that encode Reps (AntCM10–12) were identified in this sample set, all from a single Chinstrap colony in the South Shetland Islands, where two genotypes, those which only contain a *rep* gene (*rep*: 963–1335 nts), were found in multiple individuals. The other genotype, AntCM11, found in a single Chinstrap Penguin, contained a *cp* gene (960 nts), an unknown ORF, and a partial *rep* (459 nts).

Among the AntVs (6 species/12 genotypes), each genotype was only detected in a single host species, with five only found in Chinstrap Penguins, one in Adélie Penguins, one in Gentoo Penguins, and five in King Penguins. Each Antarctic virus sequence was only found at a single site, though 8/12 genotypes were found in multiple individuals at those sites (Figure 2). AntV3 has five genotypes, two of which (genotype III and IV) were only found in King Penguins from South Georgia, sharing 82.5–94.5% genome-wide identity with the three genotypes (genotype I, II and V) found only in Chinstrap Penguins from Deception Island (Supplementary Data 1), sites separated by approximately ~1700 km of the Southern Ocean (Figure 1, Supplementary Table S1). This suggests that this virus is broadly distributed across the Scotia Arc.

Cases of parent-chick viral genotype sharing were found in each Pygoscelid species. AntV1 genotype II was found in one Chinstrap Penguin adult/chick pair at Georges Point (Western Antarctic Peninsula), whereas AntV6 was shared in one Adélie Penguin adult/chick pair on Booth Island (Western Antarctic Peninsula). AntV2 was identified in five Gentoo Penguin individuals from Yankee Harbor in the South Shetland Islands, including an adult/chick pair (Figure 2, Table 1, and Supplementary Data 1). King Penguin chicks had already fledged during sampling, thus parentage could not be determined in this sample set. In those cases where penguin parents and their chicks both share the same viruses, future studies have to be conducted to observe if those were vertically or horizontally transmitted.

Co-occurrence of these novel AntVs and AntCMs was noted in 14/32 Chinstrap Penguins (2–6 sequences per individual), while 11/20 King Penguins carried two or more viral sequences (maximum of 3 sequences in one chick) (Figure 2, Supplementary Table S1). At this time, we are unable to discern whether the presence or co-occurrence of one or more AntVs or AntCMs indicates that these infect penguins, cause decreased fitness in the hosts, or are penguin-associated through trophic transfer.

### 3.3. Analyses of the Replication-Associated Protein

The hallmarks of the Rep protein are the endonuclease and helicase domains that are involved in the initiation of replication in ssDNA viruses, and to some extent, in bacterial plasmids (reviewed in Kazlauskas et al. [35] and Rosario et al. [60]). The Reps encoded by the AntVs (*n* = 40) have the rolling circle replication (RCR) endonuclease domain and the super family 3 (SF3) helicase motifs (Table 3). Thirteen of the 14 and AntCMs also have the RCR domain and SF3 motifs. AntCM11, which has a partial Rep, is missing the RCR domain and the Arg finger domain (Table 3).

**Figure 3:**
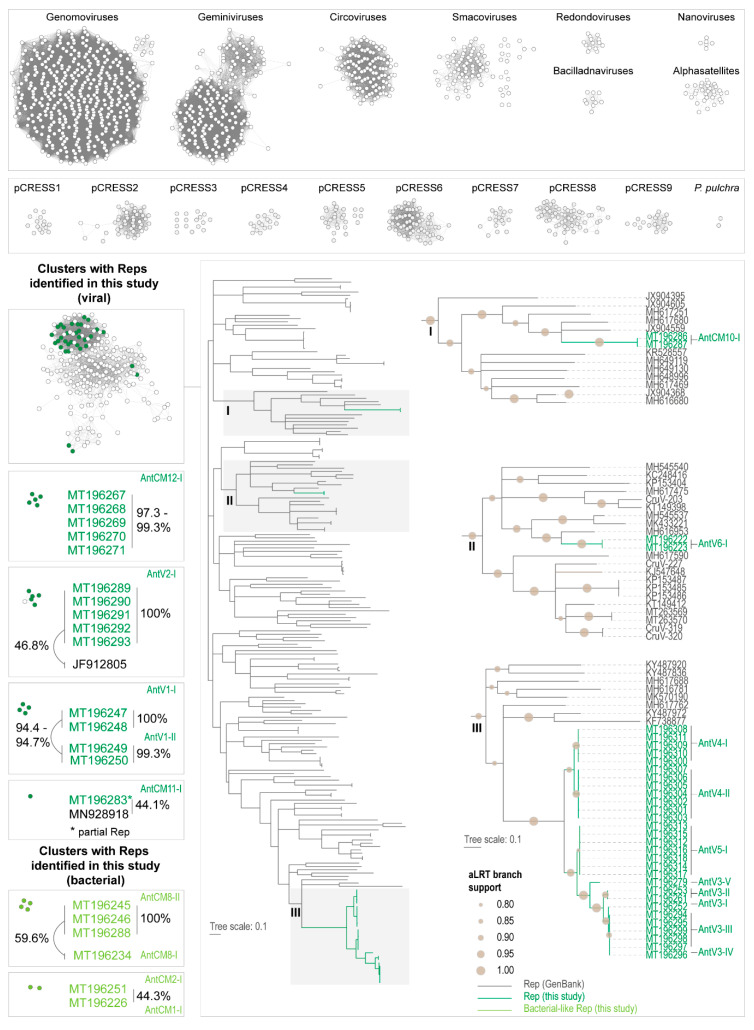
Sequence similarity network (SSN) analysis and Maximum likelihood (ML) phylogenetic tree of the Rep amino acid sequences identified in this study compared with those of known viruses in classified families and plasmids identified by Kazlauskas et al. [35]. The ML phylogenetic tree based on the SSN is mid-point rooted, and branches with aLRT support < 0.8 have been collapsed. The Reps identified in this study are highlighted in green and relevant clades of the tree are shown in the expanded view. Sequences with taxa names CruV-203, -227, -319, and -320 are from metagenomic derived sequences reported in de la Higuera et al. [64] and do not have accession #s assigned. The level of branch support (>0.8) is depicted as different sized shaded circles.

Since we identified six AntCMs with Reps that are most closely related to those of plasmids, we included a suite of plasmids grouped by Kazlauskas et al. [35] for clustering the Reps using a sequence similarity network (SSN) based approach. We have found in the past [26,61,62] that an SSN analysis using EST-EFI [36,37] with a network threshold of 60 allows for reasonable family level clusters for families in the phylum *Cressdnaviricota* [27]. Based on the sequence similarity network analysis of the Rep amino acid sequences (Figure 3), none of the AntV Reps cluster with known viral sequences. A vast majority of the AntV Reps (*n* = 33) are part of a large cluster of Reps (*n* = 213). The 33 AntV Reps within this cluster are distributed within 3 clades in the ML phylogenetic tree (RtREV+I+G+F amino acid substitution model; Figure 3). One of the clades (Clade I in Figure 3) includes Reps of AntCM10. The second clade (Clade II in Figure 3) includes Reps of AntV6. The Reps of AntV3, -4 and -5 are represented in Clade III (Figure 3), forming a well-supported clade and share >72% amino acid identity. The Reps of AntCV3, -4 and-5 share < 60% amino acid identity with other Reps in this cluster. AntCV2 Reps, which share 100% amino acid identity, cluster with a Rep of Copepod LaCopCV (JF912805) sampled in Tampa Bay (Florida, USA) [63] sharing 46.8% amino acid identity. AntCV1 Reps cluster together: Those that are genotype I share 94.4–94.7% amino acid identity with genotype II (Figure 3). AntCM12 Reps cluster together sharing >97.3% amino acid identity. The AntCM11 with a partial Rep is a singleton sharing 44.1% amino acid identity to a Rep of a virus (MN928918) from a cloacal swab of an Indian blue peafowl (*Pavo cristatus*).

The plasmid-like Reps of AntCM1, -2 and -8 do not cluster with the plasmids (pCRESS) grouped by [35] and form two clusters, one of two Reps (AntCM1 and -2) sharing 44% pairwise amino acid identity and one of four Reps with AntCM8 genotype I Reps sharing 100% identity and collectively they share 59.6% identity with that of AntCM8 genotype II (Figure 3).

### 3.4. Analyses of the Capsid Protein

In general, for viruses in the phylum *Cressdnaviricota*, the CPs are more diverse than the Reps. Furthermore, the CPs of geminiviruses share homology to those of albetoviruses (linear ssRNA(+) genomes) [65], and a group of unclassified cressdnavirues have CPs that are homologous to those of tombusviruses (linear ssRNA(+) genomes) [64,66,67,68].

**Figure 4:**
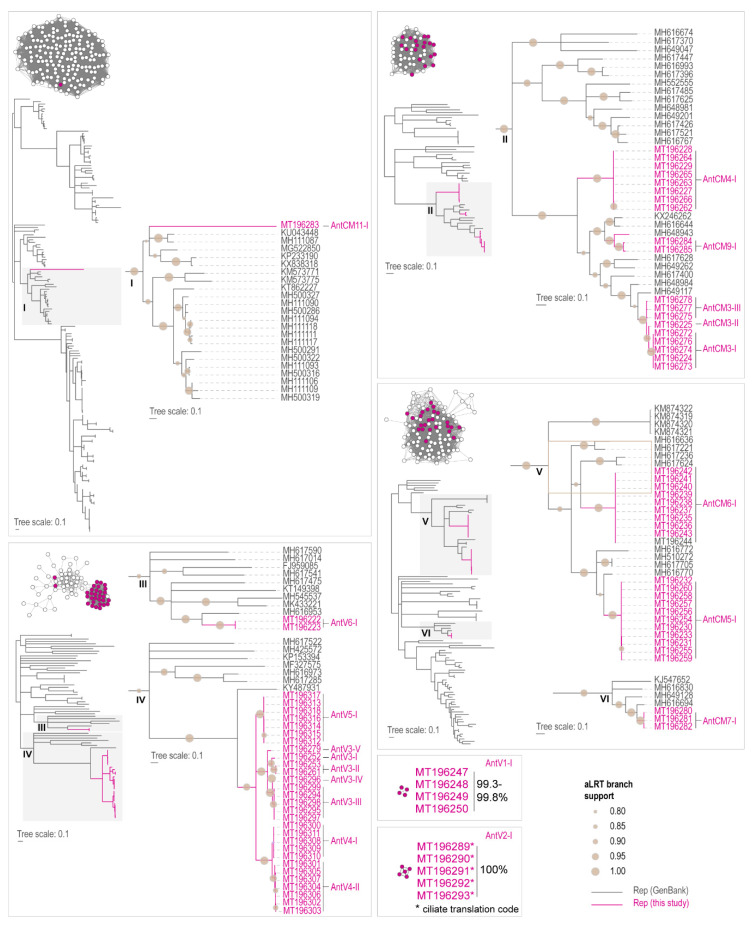
Sequence similarity network (SSN) analysis and Maximum likelihood (ML) phylogenetic tree of the CP amino acid sequences that fall within specific networks that encompass sequences identified in this study (highlighted in purple). The ML phylogenetic trees based on the SSNs are mid-point rooted, and branches with aLRT support < 0.8 have been collapsed. The level of branch support (>0.8) is depicted as different sized shaded circles.

The SSN analysis of the CPs reveals that AntCM11 CP clusters with those of smacoviruses and smacovirus-like CPs (*n* = 175) sharing < 33% identity (Figure 4). In a ML phylogenetic tree (amino acid substitution model LG+I+G+F), AntCM11 CP is part of a well-supported clade (Clade I, Figure 4). The remaining 83 CPs identified in this study cluster with none of the classified viral groups.

The CPs of AntCM3, -4 and -9 are part of a cluster of 57 CPs sharing 46–57% amino acid identity. In the ML phylogenetic tree (amino acid substitution model rtRev+I+G+F) these CPs are part of a well-supported clade (clade II; Figure 4). The CP of AntCM3 is most closely related (sharing 76–79% amino acid identity) to that of MH648984 identified in a seabass tissue [47]. AntCM4 CP shares ~49% amino acid identity with the CP of MH649117 from a seabass tissue, whereas AntCM9 shares 69–77% amino acid identity with MH648943 (seabass tissue), MH616644 (red snapper tissue) and KX246262 (sea cucumber sample) [47,69].

The CPs of AntV3, -4, -5 and -6 are part of a cluster of 77 CPs but the CPs of AntV6 (Clade III) are part of a distinct clade in the ML phylogenetic tree (amino acid substitution model LG+I+G+F) that is different from the one of AntV3, -4 and -5 (Figure 4). AntCV6 CPs share ~40% amino acid identity with the CP of MH616953 from rainbow trout tissue [47]. The CPs of AntV3, -4, and -5 share >61% amino acid identity and less that 40% identity with other CPs within the cluster.

The CPs of AntCM5, -6 and -7 are part of the same cluster composed of 108 CPs and form two distinct clades in the ML phylogenetic tree (amino acid substitution model LG+I+G+F) with AntCM5 and -6 CPs being part of the same clade (clade V; Figure 4) sharing ~40% amino acid identity. AntCM5 CP shares the highest amino acid identity (~73%) with the CPs of MH510272 and MH617705 both from seabass tissue [47]. On the other hand, the CPs of AntCM6 share the highest amino acid identity (~46%) with the CPs of MH617236 and MH617624 identified in abalone and red snapper tissue, respectively [47]. When compared to other CPs in this cluster, CPs of AntCM7 are part of a smaller clade (clade VI; Figure 4), sharing the highest amino acid identity of 77.6% with the CP of MH616694 and MH617624 of a viral sequence identified in abalone tissue [47].

AntV1 and -2 CPs form distinct clusters, each sharing 99.3–99.8 and 100% pairwise identity, respectively (Figure 4). The CPs of AntV2 (from translation using the ciliate translation table) share the highest identity (35%) with CP-like element MK012509 from crucian carp tissue, whereas those of AntCV1 share ~27% with a CP-like element of MH648877 from seabass tissue [47]. Although the CPs from this study have a high degree of sequence diversity, it is interesting to note they are more closely related to other CPs of viruses identified in the marine ecosystem in which penguins play important roles.

### 3.5. Recombination Analysis

**Figure 5:**
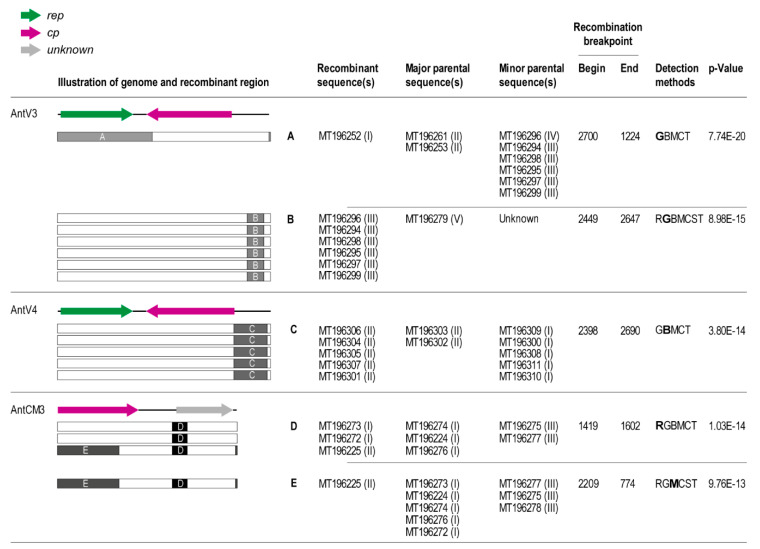
Summary of the recombination detected by RDP4. The methods used to detect recombination are RDP (R) GENCONV (G), BOOTSCAN (B), MAXCHI (M), CHIMERA (C), SISCAN (S), and 3SEQ (T). The method with the highest p-value for each recombination event is bolded. For each genome, a graphic on the left indicates the recombination event with its breakpoint location within the genome. Roman numerals in parenthesis after accession numbers indicate genotype.

The AntVs and AntCMs were aligned using MAFFT [39] at a species level, and these were used to identify evidence of recombination using RDP4 v.4.97 [45]. We identified evidence of recombination in genomes of AntV3 and -4, and AntCM3 (Figure 5). For AntV3, two recombination events were identified, one spanning ~46% of the genome, with genotype I being the recombinant, with the recombinant region derived from genotypes III and IV. The AntV3 genotype III has a 198 nts recombinant region from the non-coding region of an unknown/unsampled viral sequence. Five variants of AntV4 genotype II have a ~290 nts recombinant region in the non-coding region derived from AntV4 genotype I. AntCM3 genotype II has two recombinant regions, both derived from genotype III and in total they account for 46% of the sequence. The second smaller recombinant region of ~180 nts is found in two AntCM3 genotype I variants as well (Figure 5). Evidence of recombination between these viruses and circular molecules indicates host commonality.

## 4. Concluding Remarks

The overall knowledge of penguin-associated viruses is limited, and thus it is not surprising that penguin disease understanding also remains limited to observations of mass mortality events, case reports, and veterinary knowledge obtained from captive animals, reviewed in Smeele et al. [12], Barbosa and Palacios [70], Clarke and Kerry [71], Woods et al. [72], and Grimaldi et al. [73]. Advances in high-throughput sequencing have shown that penguins are associated with a number of (pathogenic or non-pathogenic) viruses not previously detected through serological means [59,74,75,76,77,78,79,80,81]. Studies such as these to directly detect nucleic acid presence in animal hosts add to our baseline knowledge, which still remains constrained by sampling effort.

In this study, the viruses and circular molecules that we identify are novel, and thus we are unable to distinguish whether penguins serve as the hosts for replication of these viruses, as they may enter the penguin gastrointestinal tract through prey consumption, indirectly through the consumption of phytoplankton or zooplankton by penguin prey, or through ingestion of ocean water. Based on the CP network analysis, it does appear that some of the CPs identified in this study have homologs in sequences derived from various marine fish. Further investigation into viral presence in other mesopredators, such as flying seabirds and pinnipeds, along with cetaceans, may help elucidate whether relationships exist among these molecules. Coupled with long-term monitoring studies of individual animals and sites in the rapidly changing Antarctic, detection of potential disease-causing agents will inform conservation management and biosecurity.

## Figures and Tables

**Table 1:** Table of detection of viral-like circular molecules (AntCM) and viruses (AntV) across sampled host species and sites based on recovery of complete sequences by PCR, cloning, and Sanger sequencing. Total sample size n (Adults/Chicks) shown in the header, while the number of individuals in which a given genotype was detected (Adults/Chicks) shown within the table. Cases where a single individual had two sequences of a single genotype are indicated with an asterisk (*). Regions: CWAP = Central Western Antarctic Peninsula, NEAP = Northeast Antarctic Peninsula, SG = South Georgia, SH = South Shetland Islands.

		Penguin Species										
		Adélie Penguin		Chinstrap Penguin				Gentoo Penguin				King Penguin
	Site, Region	Kinnes Cove, NEAP	Booth Is., CWAP	Half Moon Is., SH	Baily Head, SH	Georges Point, CWAP	Booth Is., CWAP	Yankee Harbor, SH	Georges Point, CWAP	Booth Is., CWAP	Moot Point, CWAP	St. Andrew’s Bay, SG
Viral Molecule	GenBank Accession(s)	*n* = 14 Adults = 7 Chicks = 7	*n* = 3 Adults = 2 Chicks = 1	*n* = 10 Adults=10 Chicks = 0	*n* = 10 Adults = 5 Chicks = 5	*n* = 6 Adults = 3 Chicks = 3	*n* = 6 Adults = 3 Chicks = 3	*n* = 9 Adults = 6 Chicks = 3	*n* = 6 Adults = 3 Chicks = 3	*n* = 6 Adults = 3 Chicks = 3	*n* = 5 Adults = 4 Chicks = 1	*n* = 20 Adults = 10 Chicks = 10
AntCM_1_I	MT196226				1/0							
AntCM_2_I	MT196251			1/0								
AntCM_3_I	MT196224, MT196272–MT196276			4/0	1/0							
AntCM_3_II	MT196225				1/0							
AntCM_3_III	MT196275, MT196277–MT196278			3/0								
AntCM_4_I	MT196227–MT196229, MT196262–MT196266			5/0	3/0							
AntCM_5_I	MT196230–MT196233, MT196254–MT196260			7/0	4/0							
AntCM_6_I	MT196235–MT196244				5/5							
AntCM_7_I	MT196280–MT196282			3/0								
AntCM_8_I	MT196234				1/0							
AntCM_8_II	MT196245–MT196246, MT196288						2*/0			1/0		
AntCM_9_I	MT196284–MT196285			2/0								
AntCM_10_I	MT196286–MT196287			2/0								
AntCM_11_I	MT196283			1/0								
AntCM_12_I	MT196267–MT196271			5/0								
AntV_1_I	MT196247–MT196248					2/0						
AntV_1_II	MT196249–MT196250					1/1						
AntV_2_I	MT196289–MT196293							4/1				
AntV_3_I	MT196252			1/0								
AntV_3_II	MT196253 MT196261			1*/0 1*/0								
AntV_3_III	MT196294–MT196295, MT196297–MT196299											1/4
AntV_3_IV	MT196296											0/1
AntV_3_V	MT196279			1/0								
AntV_4_I	MT1962											5/0
AntV_4_II	MT196301–MT196207											1/6
AntV_5_I	MT196312–MT196218											5/2
AntV_6_I	MT196222–MT196223		1/1									

**Table 2:** Summary of the BLASTn result of the representative sequences of AntCMs and AntVs.

Virus/ Circular Molecule	Top BLASTn Hit Accession	Source	Query Cover	*E*-Value	Identity
AntCM1	-		-	-	-
AntCM2	-		-	-	-
AntCM3	MH648984	Seabass tissue	86%	4.00 × 10^−167^	73.86%
AntCM4	JX904537	Sea water (Saanich Inlet)	48%	0	79.50%
AntCM5	MH510272	Seabass tissue	43%	4.00 × 10^−84^	69.79%
AntCM6	MH616824	Abalone tissue	24%	2.00 × 10^−42^	72.02%
AntCM7	MH616694	Abalone tissue	41%	3.00 × 10^−110^	72.54%
AntCM8	-		-	-	-
AntCM9	JX904537	Sea water (Saanich Inlet)	87%	0	88.53%
AntCM10	JX904559	Sea water (Saanich Inlet)	53%	1.00 × 10^−76^	68.88%
AntCM11	-		-	-	-
AntCM12	MH649197	Red snapper tissue	71%	8.00 × 10^−170^	70.75%
AntV1	-		-	-	-
AntV2	-		-	-	-
AntV3	-		-	-	-
AntV4	-		-	-	-
AntV5	-		-	-	-
AntV6	-		-	-	-

**Table 3:** Summary of the rolling circle replication (RCR) endonuclease motifs (Motif I, II, II) and SF3 helicase motifs (Walker A, Walker B, and Motif C) and Arg finder motif of the Reps identified in this study.

Virus/ Circular Molecule	Genotype	Isolate	Accession #	Motif I	Motif II	Motif III	Walker A	Walker B	Motif C	Arg Finger
AntV1	I	CPGEORsw001Ad	MT196247	CVTINN	HVQM	WADQEYCFK	GPAGCGKNKLTT	LMHEY	VFTTN	AFWRRI
AntV1	I	CPGEORsw002Ad	MT196248	CVTINN	HVQM	WADQEYCFK	GPAGCGKNKLTT	LMHEY	VFTTN	AFWRRI
AntV1	II	CPGEORsw003Ad	MT196249	CVTINN	HVQM	WADQEYCFK	GPAGCGKNKLTT	LMHEY	VFTTN	AFWRRI
AntV1	II	CPGEORsw003Ch	MT196250	CVTINN	HVQM	WADQEYCFK	GPAGCGKNKLTT	LMHEY	VFTTN	AFWRRI
AntV2	I	GPYANKsw001Ad	MT196289	SITYNN	HFQC	MAAFKYCQK	GVPGSGKSYQAR	IIEEM	IVTSN	AIKRRF
AntV2	I	GPYANKsw001Ch	MT196290	SITYNN	HFQC	MAAFKYCQK	GVPGSGKSYQAR	IIEEM	IVTSN	AIKRRF
AntV2	I	GPYANKsw002Ad	MT196291	SITYNN	HFQC	MAAFKYCQK	GVPGSGKSYQAR	IIEEM	IVTSN	AIKRRF
AntV2	I	GPYANKsw003Ad	MT196292	SITYNN	HFQC	MAAFKYCQK	GVPGSGKSYQAR	IIEEM	IVTSN	AIKRRF
AntV2	I	GPYANKsw004Ad	MT196293	SITYNN	HFQC	MAAFKYCQK	GVPGSGKSYQAR	IIEEM	IVTSN	AIKRRF
AntV3	I	CPHALFsw004Ad	MT196252	CFTLNN	HWQG	QQAIDYCKK	GETGTGKSRKLW	ALEEW	IVTSN	PLKRRF
AntV3	II	CPHALFsw005Ad	MT196253	CFTLNN	HWQG	TQAIDYCKK	GETGTGKSRKLW	ALEEW	IVTSN	PLKRRF
AntV3	II	CPHALFsw005Ad	MT196261	CFTLNN	HWQG	TQAIDYCKK	GETGTGKSRKLW	ALEEW	IVTSN	PLKRRF
AntV3	III	KPSTAsw010Ad	MT196294	CFTLNN	HWQG	QQAIDYCKK	GETGTGKSRKLW	ALEEW	IVTSN	PLKRRF
AntV3	III	KPSTAsw014Ch	MT196295	CFTLNN	HWQG	QQAIDYCKK	GETGTGKSRKLW	ALEEW	IVTSN	PLKRRF
AntV3	III	KPSTAsw016Ch	MT196297	CFTLNN	HWQG	QQAIDYCKK	GETGTGKSRKLW	ALEEW	IVTSN	PLKRRF
AntV3	III	KPSTAsw018Ch	MT196298	CFTLNN	HWQG	QQAIDYCKK	GETGTGKSRKLW	ALEEW	IVTSN	PLKRRF
AntV3	III	KPSTAsw019Ch	MT196299	CFTLNN	HWQG	QQAIDYCKK	GETGTGKSRKLW	ALEEW	IVTSN	PLKRRF
AntV3	IV	KPSTAsw015Ch	MT196296	CFTLNN	HWQG	QQAIDYCKK	GETGTGKSRKLW	ALEEW	IVTSN	PLKRRF
AntV3	V	CPHALFsw003Ad	MT196279	VFTINN	HWQG	KQAIDYCKK	GATGTGKSRKLW	ALEEW	IVTSN	PLKRRF
AntV4	I	KPSTAsw001Ad	MT196300	CFTINN	HWQG	QQAIDYCKK	GGTGTGKSRKLW	AIEEW	IVTSN	PLKRRF
AntV4	I	KPSTAsw002Ad	MT196308	CFTINN	HWQG	QQAIDYCKK	GGTGTGKSRKLW	AIEEW	IVTSN	PLKRRF
AntV4	I	KPSTAsw008Ad	MT196309	CFTINN	HWQG	QQAIDYCKK	GGTGTGKSRKLW	AIEEW	IVTSN	PLKRRF
AntV4	I	KPSTAsw009Ad	MT196310	CFTINN	HWQG	QQAIDYCKK	GGTGTGKSRKLW	AIEEW	IVTSN	PLKRRF
AntV4	I	KPSTAsw010Ad	MT196311	CFTINN	HWQG	QQAIDYCKK	GGTGTGKSRKLW	AIEEW	IVTSN	PLKRRF
AntV4	II	KPSTAsw004Ad	MT196301	CFTINN	HWQG	QQAIDYCKK	GETGTGKSRKLW	AIEEW	IVTSN	PLKRRF
AntV4	II	KPSTAsw014Ch	MT196302	CFTINN	HWQG	QQAIDYCKK	GETGTGKSRKLW	AIEEW	IVTSN	PLKRRF
AntV4	II	KPSTAsw015Ch	MT196303	CFTINN	HWQG	QQAIDYCKK	GETGTGKSRKLW	AIEEW	IVTSN	PLKRRF
AntV4	II	KPSTAsw016Ch	MT196304	CFTINN	HWQG	QQAIDYCKK	GETGTGKSRKLW	AIEEW	IVTSN	PLKRRF
AntV4	II	KPSTAsw017Ch	MT196305	CFTINN	HWQG	QQAIDYCKK	GETGTGKSRKLW	AIEEW	IVTSN	PLKRRF
AntV4	II	KPSTAsw018Ch	MT196306	CFTINN	HWQG	QQAIDYCKK	GETGTGKSRKLW	AIEEW	IVTSN	PLKRRF
AntV4	II	KPSTAsw019Ch	MT196307	CFTINN	HWQG	QQAIDYCKK	GETGTGKSRKLW	AIEEW	IVTSN	PLKRRF
AntV5	I	KPSTAsw001Ad	MT196312	CFTLNN	HWQG	KQAIDYCKK	GATGTGKSQKLW	AIEEW	IVTSN	PLKRRF
AntV5	I	KPSTAsw002Ad	MT196313	CFTLNN	HWQG	KQAIDYCKK	GATGTGKSQKLW	AIEEW	IVTSN	PLKRRF
AntV5	I	KPSTAsw004Ad	MT196314	CFTLNN	HWQG	KQAIDYCKK	GATGTGKSQKLW	AIEEW	IVTSN	PLKRRF
AntV5	I	KPSTAsw008Ad	MT196315	CFTLNN	HWQG	KQAIDYCKK	GATGTGKSQKLW	AIEEW	IVTSN	PLKRRF
AntV5	I	KPSTAsw009Ad	MT196316	CFTLNN	HWQG	KQAIDYCKK	GATGTGKSQKLW	AIEEW	IVTSN	PLKRRF
AntV5	I	KPSTAsw018Ch	MT196317	CFTLNN	HWQG	KQAIDYCKK	GATGTGKSQKLW	AIEEW	IVTSN	PLKRRF
AntV5	I	KPSTAsw020Ch	MT196318	CFTLNN	HWQG	KQAIDYCKK	GATGTGKSQKLW	AIEEW	IVTSN	PLKRRF
AntV6	I	APBOOTsw001Ad	MT196222	VFTWNN	HLQG	QECDKYCRK	GESGCGKTRAVH	LLDDI	IVTSQ	ALLRRF
AntV6	I	APBOOTsw001Ch	MT196223	VFTWNN	HLQG	QECDKYCRK	GESGCGKTRAVH	LLDDI	IVTSQ	ALLRRF
AntCM1	I	CPBAILsw002Ad	MT196226	IVTLPA	HWQI	QQAYEYVTK	GPTRVGKSRNVV	VLDEF	WVVSN	AFEARF
AntCM2	I	CPHALFsw001Ad	MT196251	LLTLPA	HWQL	QQVYDYVTK	GPTGVGKTRHIY	VLDEF	WIVSN	AFRRRL
AntCM8	I	CPBAILsw001Ad	MT196234	FLTISA	HYHA	AKSIKYLKK	GAAGGGKSTLAR	VYDEF	IIVSN	AFKRRI
AntCM8	II	CPBOOTsw002Ad	MT196245	FITISG	HYHI	HDVLKYIQK	GVPGGGKSTLAR	IFDEF	IIASN	AFKRRI
AntCM8	II	CPBOOTsw002Ad	MT196246	FITISG	HYHI	HDVLKYIQK	GVPGGGKSTLAR	IFDEF	IIASN	AFKRRI
AntCM8	II	GPBOOTsw003Ad	MT196288	FITISG	HYHI	HDVLKYIQK	GVPGGGKSTLAR	IFDEF	IIASN	AFKRRI
AntCM10	I	CPHALFsw008Ad	MT196286	MFTINN	HYQG	KQAVDYVSK	GKPGTGKSHQAR	ILDDL	VVTSN	AINRRF
AntCM10	I	CPHALFsw010Ad	MT196287	MFTINN	HYQG	KQAVDYVSK	GKPGTGKSHQVR	ILDDL	VVTSN	AINRRF
AntCM11	I	CPHALFsw005Ad	MT196283	-	-	-	DYEGNKGKSWLSR	IIDVP	MVITN	-
AntCM12	I	CPHALFsw003Ad	MT196267	FITIPQ	HYHI	NAVITYIKK	GKPNSGKSFMFN	CWDIP	IVFAN	LPQGRT
AntCM12	I	CPHALFsw004Ad	MT196268	FITIPQ	HYHI	PAVITYIKK	GRPNSGKSFMFN	CWDIP	IVFAN	LPKGRT
AntCM12	I	CPHALFsw008Ad	MT196269	FITIPQ	HYHI	PAVITYIKK	GRPNSGKSFMFN	CWDIP	IVFAN	LPKGRT
AntCM12	I	CPHALFsw009Ad	MT196270	FITIPQ	HYHI	PAVITYIKK	GRPNSGKSFMFN	CWDIP	IVFAN	LPKGRT
AntCM12	I	CPHALFsw010Ad	MT196271	FITIPQ	HYHI	NAVITYIKK	GKPNSGKSFMFN	CWDIP	IVFAN	LPKGRT

## Supplementary Materials

The following are available online at https://www.mdpi.com/1999-4915/12/9/1029/s1, Supplementary Table S1. Sampling site details and coordinates for four penguin species, along with cloacal swabs obtained in terms of total numbers of individuals, and breakdown by adults and chicks; Supplementary Table S2: Summary of the reads from the HTS; Supplementary Table S3: Summary of AntV and AntCM sequences identified in this study together with the sampling date, location, host, and the primer pairs used to recover the sequences. Supplementary Data 1: Details of cloacal swab samples of the four penguin species. Supplementary Data 2: Pairwise identity matrix for determination of AntV and AntCM species and genotypes.
