# Supplementary material for: Identification and Distribution of Novel Cressdnaviruses and Circular Molecules in Four Penguin Species in South Georgia and the Antarctic Peninsula
Source: Viruses. 2020 Sep 16;12(9):1029. doi: 10.3390/v12091029 (PMC7551938; doi:10.3390/v12091029)
Supplement: Supplementary file 1 [file viruses-12-01029-s001.zip › Supplementary_Table_S1.docx]

**Supplementary Table S1.** Sampling site details and coordinates for four penguin species, along with cloacal swabs obtained in terms of total numbers of individuals, and breakdown by adults and chicks.

| **Colony and Antarctic Site Inventory Subarea** | **Coordinates** | **Penguin Species (Individuals Sampled)** | **Adults Sampled** | **Chicks Sampled** |
| --- | --- | --- | --- | --- |
| St. Andrew’s Bay (STA), South Georgia | 54°26’S,  36°11’W | King (20) | 10 | 10 |
| Yankee Harbor (YANK), Greenwich Island, South Shetland Islands | 62˚32’S, 59˚47’W | Gentoo (9) | 6 | 3 |
| Half Moon Island (HALF), South Shetland Islands | 62˚36’S, 59˚55’W | Chinstrap (10) | 10 | - |
| Baily Head (BAIL), Deception Island, South Shetland Islands | 62°58’S, 60°30’W | Chinstrap (10) | 5 | 5 |
| Kinnes Cove (KINN, Madder Cliff), Joinville Island, Northeast Antarctic Peninsula | 63°18’S, 56°29’W | Adélie (14) | 7 | 7 |
| Georges Point (GEOR), Ronge Island, Central Western Antarctic Peninsula | 64°40’S, 62°40’W | Chinstrap (6)  Gentoo (6) | 3  3 | 3  3 |
| Port Charcot (BOOT), Booth Island, Central Western Antarctic Peninsula | 65°05’S, 64°00’W | Adélie (3)  Chinstrap (6)  Gentoo (6) | 2  3  3 | 1  3  3 |
| Moot Point (MOOT), Central Western Antarctic Peninsula | 65°12’S, 64°06’W | Gentoo (5) | 4 | 1 |
| **Total by Species** | | **Adélie (17)**  **Chinstrap (32)**  **Gentoo (26)**  **King (20)** | **9**  **21**  **16**  **10** | **8**  **11**  **10**  **10** |
