# Supplementary material for: Identification and Distribution of Novel Cressdnaviruses and Circular Molecules in Four Penguin Species in South Georgia and the Antarctic Peninsula
Source: Viruses. 2020 Sep 16;12(9):1029. doi: 10.3390/v12091029 (PMC7551938; doi:10.3390/v12091029)
Supplement: Supplementary file 1 [file viruses-12-01029-s001.zip › Supplementary_Table_S2.docx]

| **Sample ID** | **Penguin species** | **Location** | **SRA accession #** | **Raw reads** | **Trimmed & quality reads** | **Contigs >500nts** |
| --- | --- | --- | --- | --- | --- | --- |
| APBOOT | Adélie penguin | Port Charcot (BOOT), Booth Island, Central Western Antarctic Peninsula | SRR12597828 | 23,448,774 | 23,426,426 | 20617 |
| APK | Adélie penguin | Kinnes Cove (KINN, Madder Cliff), Joinville Island, Northeast Antarctic Peninsula | SRR12597829 | 24,312,866 | 24,292,264 | 18220 |
| CPBAIL | Chinstrap penguin | Baily Head (BAIL), Deception Island, South Shetland Islands | SRR12597834 | 35,362,402 | 35,333,603 | 8162 |
| CPBOOT | Chinstrap penguin | Port Charcot (BOOT), Booth Island, Central Western Antarctic Peninsula | SRR12597836 | 30,626,698 | 30,598,410 | 16866 |
| CPGEOR | Chinstrap penguin | Georges Point (GEOR), Ronge Island, Central Western Antarctic Peninsula | SRR12597833 | 29,955,832 | 29,932,936 | 15445 |
| CPHALF | Chinstrap penguin | Half Moon Island (HALF), South Shetland Islands | SRR12597832 | 36,290,256 | 36,262,045 | 24963 |
| GPBOOT | Gentoo penguin | Port Charcot (BOOT), Booth Island, Central Western Antarctic Peninsula | SRR12597827 | 20,412,398 | 20,392,560 | 8764 |
| GPGEOR | Gentoo penguin | Georges Point (GEOR), Ronge Island, Central Western Antarctic Peninsula | SRR12597835 | 24,158,508 | 24,134,800 | 6075 |
| GPMO | Gentoo penguin | Moot Point (MOOT), Central Western Antarctic Peninsula | SRR12597831 | 27,663,634 | 27,639,078 | 9712 |
| GPY | Gentoo penguin | Yankee Harbor (YANK), Greenwich Island, South Shetland Islands | SRR12597830 | 25,634,528 | 25,610,169 | 15618 |
| KPSTA | King penguin | St. Andrew’s Bay (STA), South Georgia | SRR12597837 | 33,248,072 | 33,215,714 | 11453 |
