# Supplementary material for: Identification and Distribution of Novel Cressdnaviruses and Circular Molecules in Four Penguin Species in South Georgia and the Antarctic Peninsula
Source: Viruses. 2020 Sep 16;12(9):1029. doi: 10.3390/v12091029 (PMC7551938; doi:10.3390/v12091029)
Supplement: Supplementary file 1 [file viruses-12-01029-s001.zip › Supplementary_Table_S3.docx]

| **Accession** | **Virus / circular**  **molecule** | **Genotype** | **Isolate** | **Collection**  **date** | **Location** | **Host** |  |
| --- | --- | --- | --- | --- | --- | --- | --- |
| MT196247 | AntV1 | I | CPGEORsw001Ad | 6-Jan-16 | Georges Point, West Antarctic peninsula | *Pygoscelis antarcticus* | F: 5'-CATAAATCATTTTGTGTTACGATCAACAACTACAC-3'  R: 5'-ACTCATCTTGAAAGAAAAGTGGTGGTCTATATTAC-3' |
| MT196248 | AntV1 | I | CPGEORsw002Ad | 6-Jan-16 | Georges Point, West Antarctic peninsula | *Pygoscelis antarcticus* | F: 5'-CATAAATCATTTTGTGTTACGATCAACAACTACAC-3';  R: 5'-ACTCATCTTGAAAGAAAAGTGGTGGTCTATATTAC-3' |
| MT196249 | AntV1 | II | CPGEORsw003Ad | 6-Jan-16 | Georges Point, West Antarctic peninsula | *Pygoscelis antarcticus* | F: 5'-CATAAATCATTTTGTGTTACGATCAACAACTACAC-3';  R: 5'-ACTCATCTTGAAAGAAAAGTGGTGGTCTATATTAC-3' |
| MT196250 | AntV1 | II | CPGEORsw003Ch | 6-Jan-16 | Georges Point, West Antarctic peninsula | *Pygoscelis antarcticus* | F: 5'-CATAAATCATTTTGTGTTACGATCAACAACTACAC-3'  R: 5'-ACTCATCTTGAAAGAAAAGTGGTGGTCTATATTAC-3' |
| MT196289 | AntV2 | I | GPYANKsw001Ad | 17-Jan-16 | Booth Island, West Antarctic peninsula | *Pygoscelis papua* | F: 5'-GCTAATAATTCAAATAATGGTTTACCGTCG-3'  R: 5'-AGTTATATTTCACAAAACCCCCTCTTATAT-3' |
| MT196290 | AntV2 | I | GPYANKsw001Ch | 17-Jan-16 | Booth Island, West Antarctic peninsula | *Pygoscelis papua* | F: 5'-GCTAATAATTCAAATAATGGTTTACCGTCG-3'  R: 5'-AGTTATATTTCACAAAACCCCCTCTTATAT-3' |
| MT196291 | AntV2 | I | GPYANKsw002Ad | 17-Jan-16 | Booth Island, West Antarctic peninsula | *Pygoscelis papua* | F: 5'-GCTAATAATTCAAATAATGGTTTACCGTCG-3'  \R: 5'-AGTTATATTTCACAAAACCCCCTCTTATAT-3' |
| MT196292 | AntV2 | I | GPYANKsw003Ad | 17-Jan-16 | Booth Island, West Antarctic peninsula | *Pygoscelis papua* | F: 5'-GCTAATAATTCAAATAATGGTTTACCGTCG-3'  R: 5'-AGTTATATTTCACAAAACCCCCTCTTATAT-3' |
| MT196293 | AntV2 | I | GPYANKsw004Ad | 17-Jan-16 | Booth Island, West Antarctic peninsula | *Pygoscelis papua* | F: 5'-GCTAATAATTCAAATAATGGTTTACCGTCG-3'  R: 5'-AGTTATATTTCACAAAACCCCCTCTTATAT-3' |
| MT196252 | AntV3 | I | CPHALFsw004Ad | 3-Jan-16 | Half Moon Island, SouthShetland | *Pygoscelis antarcticus* | F: 5'-AGATCATACTTAAGAGTCTTAACAATCCTATACGC-3'  R: 5'-TAGTCCCAATGAGATTATTCAAAAGAAGTTTTTCT-3' |
| MT196261 | AntV3 | II | CPHALFsw005Ad | 3-Jan-16 | Half Moon Island, SouthShetland | *Pygoscelis antarcticus* | F: 5'-TTCATCACCCTTAGTCTCTTCATCTAAGTTGTTTA-3'  R: 5'-TAAATTACATTTTGACTTAAATAAGTGTTGGACGT-3' |
| MT196253 | AntV3 | II | CPHALFsw005Ad | 3-Jan-16 | Half Moon Island, SouthShetland | *Pygoscelis antarcticus* | F: 5'-AGATCATACTTAAGAGTCTTAACAATCCTATACGC-3'  R: 5'-TAGTCCCAATGAGATTATTCAAAAGAAGTTTTTCT-3' |
| MT196294 | AntV3 | III | KPSTAsw010Ad | 22-Dec-15 | St. Andrews, South Georgia | *Aptenodytes patagonicus* | F: 5'-CTTTAGTCTCTTCATCTAAGTTGTTTATCACATCG-3'  R: 5'-GCGATGAATAAACTACATGTTTTGCATTTAAATAA-3' |
| MT196295 | AntV3 | III | KPSTAsw014Ch | 22-Dec-15 | St. Andrews, South Georgia | *Aptenodytes patagonicus* | F: 5'-CTTTAGTCTCTTCATCTAAGTTGTTTATCACATCG-3'  R: 5'-GCGATGAATAAACTACATGTTTTGCATTTAAATAA-3' |
| MT196297 | AntV3 | III | KPSTAsw016Ch | 22-Dec-15 | St. Andrews, South Georgia | *Aptenodytes patagonicus* | F: 5'-CTTTAGTCTCTTCATCTAAGTTGTTTATCACATCG-3'  R: 5'-GCGATGAATAAACTACATGTTTTGCATTTAAATAA-3' |
| MT196298 | AntV3 | III | KPSTAsw018Ch | 22-Dec-15 | St. Andrews, South Georgia | *Aptenodytes patagonicus* | F: 5'-CTTTAGTCTCTTCATCTAAGTTGTTTATCACATCG-3'  R: 5'-GCGATGAATAAACTACATGTTTTGCATTTAAATAA-3' |
| MT196299 | AntV3 | III | KPSTAsw019Ch | 22-Dec-15 | St. Andrews, South Georgia | *Aptenodytes patagonicus* | F: 5'-CTTTAGTCTCTTCATCTAAGTTGTTTATCACATCG-3'  R: 5'-GCGATGAATAAACTACATGTTTTGCATTTAAATAA-3' |
| MT196296 | AntV3 | IV | KPSTAsw015Ch | 22-Dec-15 | St. Andrews, South Georgia | *Aptenodytes patagonicus* | F: 5'-CTTTAGTCTCTTCATCTAAGTTGTTTATCACATCG-3'  R: 5'-GCGATGAATAAACTACATGTTTTGCATTTAAATAA-3' |
| MT196279 | AntV3 | V | CPHALFsw003Ad | 3-Jan-16 | Half Moon Island, SouthShetland | *Pygoscelis antarcticus* | F: 5'-AACTACATGTTTTACATTTAAATAAGTGTTGGACG-3'  R: 5'-TATTCATCTCCTTTAGTTTCCTCGTCTAAATTATG-3' |
| MT196300 | AntV4 | I | KPSTAsw001Ad | 22-Dec-15 | St. Andrews, South Georgia | *Aptenodytes patagonicus* | F: 5'-CACATCGACAAGTATATTGTTGACTCCCGTATTCA-3'  R: 5'-GAGGTTGTAGTTTGTATCATTGGTAAGATCAAACA-3' |
| MT196308 | AntV4 | I | KPSTAsw002Ad | 22-Dec-15 | St. Andrews, South Georgia | *Aptenodytes patagonicus* | F: 5'-CAACACAATGCCAACAACAATTCATTTTTCTAATA-3'  R: 5'-GGGAATGTATTAAATATGGGCGAGTGGGCATTAAA-3' |
| MT196309 | AntV4 | I | KPSTAsw008Ad | 22-Dec-15 | St. Andrews, South Georgia | *Aptenodytes patagonicus* | F: 5'-CAACACAATGCCAACAACAATTCATTTTTCTAATA-3'  R: 5'-GGGAATGTATTAAATATGGGCGAGTGGGCATTAAA-3' |
| MT196310 | AntV4 | I | KPSTAsw009Ad | 22-Dec-15 | St. Andrews, South Georgia | *Aptenodytes patagonicus* | F: 5'-CAACACAATGCCAACAACAATTCATTTTTCTAATA-3'  R: 5'-GGGAATGTATTAAATATGGGCGAGTGGGCATTAAA-3' |
| MT196311 | AntV4 | I | KPSTAsw010Ad | 22-Dec-15 | St. Andrews, South Georgia | *Aptenodytes patagonicus* | F: 5'-CAACACAATGCCAACAACAATTCATTTTTCTAATA-3'  F: 5'-GGGAATGTATTAAATATGGGCGAGTGGGCATTAAA-3' |
| MT196301 | AntV4 | II | KPSTAsw004Ad | 22-Dec-15 | St. Andrews, South Georgia | *Aptenodytes patagonicus* | F: 5'-CACATCGACAAGTATATTGTTGACTCCCGTATTCA-3'  R: 5'-GAGGTTGTAGTTTGTATCATTGGTAAGATCAAACA-3' |
| MT196302 | AntV4 | II | KPSTAsw014Ch | 22-Dec-15 | St. Andrews, South Georgia | *Aptenodytes patagonicus* | F: 5'-CACATCGACAAGTATATTGTTGACTCCCGTATTCA-3'  R: 5'-GAGGTTGTAGTTTGTATCATTGGTAAGATCAAACA-3' |
| MT196303 | AntV4 | II | KPSTAsw015Ch | 22-Dec-15 | St. Andrews, South Georgia | *Aptenodytes patagonicus* | F: 5'-CACATCGACAAGTATATTGTTGACTCCCGTATTCA-3'  R: 5'-GAGGTTGTAGTTTGTATCATTGGTAAGATCAAACA-3' |
| MT196304 | AntV4 | II | KPSTAsw016Ch | 22-Dec-15 | St. Andrews, South Georgia | *Aptenodytes patagonicus* | F: 5'-CACATCGACAAGTATATTGTTGACTCCCGTATTCA-3'  R: 5'-GAGGTTGTAGTTTGTATCATTGGTAAGATCAAACA-3' |
| MT196305 | AntV4 | II | KPSTAsw017Ch | 22-Dec-15 | St. Andrews, South Georgia | *Aptenodytes patagonicus* | F: 5'-CACATCGACAAGTATATTGTTGACTCCCGTATTCA-3'  R: 5'-GAGGTTGTAGTTTGTATCATTGGTAAGATCAAACA-3' |
| MT196306 | AntV4 | II | KPSTAsw018Ch | 22-Dec-15 | St. Andrews, South Georgia | *Aptenodytes patagonicus* | F: 5'-CACATCGACAAGTATATTGTTGACTCCCGTATTCA-3'  R: 5'-GAGGTTGTAGTTTGTATCATTGGTAAGATCAAACA-3' |
| MT196307 | AntV4 | II | KPSTAsw019Ch | 22-Dec-15 | St. Andrews, South Georgia | *Aptenodytes patagonicus* | F: 5'-CACATCGACAAGTATATTGTTGACTCCCGTATTCA-3'  R: 5'-GAGGTTGTAGTTTGTATCATTGGTAAGATCAAACA-3' |
| MT196312 | AntV5 | I | KPSTAsw001Ad | 22-Dec-15 | St. Andrews, South Georgia | *Aptenodytes patagonicus* | F: 5'-CCCTAACCAGATCCTTCCTATGAAATTTTATCTC-3'  R: 5'-GAAAGATCAAGCTTGTGAGTTTTTATGATCTTAA-3' |
| MT196313 | AntV5 | I | KPSTAsw002Ad | 22-Dec-15 | St. Andrews, South Georgia | *Aptenodytes patagonicus* | F: 5'-CCCTAACCAGATCCTTCCTATGAAATTTTATCTC-3'  R: 5'-GAAAGATCAAGCTTGTGAGTTTTTATGATCTTAA-3' |
| MT196314 | AntV5 | I | KPSTAsw004Ad | 22-Dec-15 | St. Andrews, South Georgia | *Aptenodytes patagonicus* | F: 5'-CCCTAACCAGATCCTTCCTATGAAATTTTATCTC-3'  R: 5'-GAAAGATCAAGCTTGTGAGTTTTTATGATCTTAA-3' |
| MT196315 | AntV5 | I | KPSTAsw008Ad | 22-Dec-15 | St. Andrews, South Georgia | *Aptenodytes patagonicus* | F: 5'-CCCTAACCAGATCCTTCCTATGAAATTTTATCTC-3'  R: 5'-GAAAGATCAAGCTTGTGAGTTTTTATGATCTTAA-3' |
| MT196316 | AntV5 | I | KPSTAsw009Ad | 22-Dec-15 | St. Andrews, South Georgia | *Aptenodytes patagonicus* | F: 5'-CCCTAACCAGATCCTTCCTATGAAATTTTATCTC-3'  R: 5'-GAAAGATCAAGCTTGTGAGTTTTTATGATCTTAA-3' |
| MT196317 | AntV5 | I | KPSTAsw018Ch | 22-Dec-15 | St. Andrews, South Georgia | *Aptenodytes patagonicus* | F: 5'-CCCTAACCAGATCCTTCCTATGAAATTTTATCTC-3'  R: 5'-GAAAGATCAAGCTTGTGAGTTTTTATGATCTTAA-3' |
| MT196318 | AntV5 | I | KPSTAsw020Ch | 22-Dec-15 | St. Andrews, South Georgia | *Aptenodytes patagonicus* | F: 5'-CCCTAACCAGATCCTTCCTATGAAATTTTATCTC-3'  R: 5'-GAAAGATCAAGCTTGTGAGTTTTTATGATCTTAA-3' |
| MT196222 | AntV6 | I | APBOOTsw001Ad | 7-Jan-16 | Booth Island, West Antarctic peninsula | *Pygoscelis adeliae* | F: 5'-AGGACAAAATATAATATTATGAGAAGGCAA-3'  R: 5'-AATACTTTTTCTATAACTACAAAACGTCGC-3' |
| MT196223 | AntV6 | I | APBOOTsw001Ch | 7-Jan-16 | Booth Island, West Antarctic peninsula | *Pygoscelis adeliae* | F: 5'-AGGACAAAATATAATATTATGAGAAGGCAA-3'  R: 5'-AATACTTTTTCTATAACTACAAAACGTCGC-3' |
| MT196226 | AntCM1 | I | CPBAILsw002Ad | 29-Dec-15 | Baily Head, Deception Island | *Pygoscelis antarcticus* | F: 5'-CCGAAACATTATAAAAACCAGAAATATCCTCAATC-3'  R: 5'-AATTTAAGGAAGAGAGATTGGAGGTTGATGAAGAT-3' |
| MT196251 | AntCM2 | I | CPHALFsw001Ad | 3-Jan-16 | Half Moon Island, SouthShetland | *Pygoscelis antarcticus* | F: 5'-GAGAGAGTATGGAAGATAAGAAGAATAGAGATACG-3'  R: 5'-ATCCCTCCAAACTTTGATACAAGATACATAAGTTG-3' |
| MT196224 | AntCM3 | I | CPBAILsw002Ad | 29-Dec-15 | Baily Head, Deception Island | *Pygoscelis antarcticus* | F: 5'-ATAGCGCAGAATAATATCATAACACCTCTCTATAA-3'  R: 5'-TGTTCCTGTCATATCCATTAGCTTAGTATATTTCT-3' |
| MT196272 | AntCM3 | I | CPHALFsw001Ad | 3-Jan-16 | Half Moon Island, SouthShetland | *Pygoscelis antarcticus* | F: 5'-TGTTCCTGTCATATCCATTAGCTTAGTATATTTCT-3'  R: 5'-ATAGCGCAGAATAATATCATAACACCTCTCTATAA-3' |
| MT196273 | AntCM3 | I | CPHALFsw003Ad | 3-Jan-16 | Half Moon Island, SouthShetland | *Pygoscelis antarcticus* | F: 5'-TGTTCCTGTCATATCCATTAGCTTAGTATATTTCT-3'  R: 5'-ATAGCGCAGAATAATATCATAACACCTCTCTATAA-3' |
| MT196274 | AntCM3 | I | CPHALFsw004Ad | 3-Jan-16 | Half Moon Island, SouthShetland | *Pygoscelis antarcticus* | F: 5'-TGTTCCTGTCATATCCATTAGCTTAGTATATTTCT-3'  R: 5'-ATAGCGCAGAATAATATCATAACACCTCTCTATAA-3' |
| MT196276 | AntCM3 | I | CPHALFsw008Ad | 3-Jan-16 | Half Moon Island, SouthShetland | *Pygoscelis antarcticus* | F: 5'-TGTTCCTGTCATATCCATTAGCTTAGTATATTTCT-3'  R: 5'-ATAGCGCAGAATAATATCATAACACCTCTCTATAA-3' |
| MT196225 | AntCM3 | II | CPBAILsw005Ad | 29-Dec-15 | Baily Head, Deception Island | *Pygoscelis antarcticus* | F: 5'-ATAGCGCAGAATAATATCATAACACCTCTCTATAA-3'  R: 5'-TGTTCCTGTCATATCCATTAGCTTAGTATATTTCT-3' |
| MT196275 | AntCM3 | III | CPHALFsw005Ad | 3-Jan-16 | Half Moon Island, SouthShetland | *Pygoscelis antarcticus* | F: 5'-TGTTCCTGTCATATCCATTAGCTTAGTATATTTCT-3'  R: 5'-ATAGCGCAGAATAATATCATAACACCTCTCTATAA-3' |
| MT196277 | AntCM3 | III | CPHALFsw009Ad | 3-Jan-16 | Half Moon Island, SouthShetland | *Pygoscelis antarcticus* | F: 5'-TGTTCCTGTCATATCCATTAGCTTAGTATATTTCT-3'  R: 5'-ATAGCGCAGAATAATATCATAACACCTCTCTATAA-3' |
| MT196278 | AntCM3 | III | CPHALFsw010Ad | 3-Jan-16 | Half Moon Island, SouthShetland | *Pygoscelis antarcticus* | F: 5'-TGTTCCTGTCATATCCATTAGCTTAGTATATTTCT-3'  R: 5'-ATAGCGCAGAATAATATCATAACACCTCTCTATAA-3' |
| MT196227 | AntCM4 | I | CPBAILsw001Ad | 29-Dec-15 | Baily Head, Deception Island | *Pygoscelis antarcticus* | F: 5'-CAGCAAAACTCATAGAATACGTAGATGATAACATA-3'  R: 5'-TTATATCATCTGTTTTCTTTTCGTATGCTAACTCT-3' |
| MT196228 | AntCM4 | I | CPBAILsw002Ad | 29-Dec-15 | Baily Head, Deception Island | *Pygoscelis antarcticus* | F: 5'-CAGCAAAACTCATAGAATACGTAGATGATAACATA-3'  R: 5'-TTATATCATCTGTTTTCTTTTCGTATGCTAACTCT-3' |
| MT196229 | AntCM4 | I | CPBAILsw004Ad | 29-Dec-15 | Baily Head, Deception Island | *Pygoscelis antarcticus* | F: 5'-CAGCAAAACTCATAGAATACGTAGATGATAACATA-3'  R: 5'-TTATATCATCTGTTTTCTTTTCGTATGCTAACTCT-3' |
| MT196262 | AntCM4 | I | CPHALFsw003Ad | 3-Jan-16 | Half Moon Island, SouthShetland | *Pygoscelis antarcticus* | F: 5'-TTATATCATCTGTTTTCTTTTCGTATGCTAACTCT-3'  R: 5'-CAGCAAAACTCATAGAATACGTAGATGATAACATA-3' |
| MT196263 | AntCM4 | I | CPHALFsw004Ad | 3-Jan-16 | Half Moon Island, SouthShetland | *Pygoscelis antarcticus* | F: 5'-TTATATCATCTGTTTTCTTTTCGTATGCTAACTCT-3'  R: 5'-CAGCAAAACTCATAGAATACGTAGATGATAACATA-3' |
| MT196264 | AntCM4 | I | CPHALFsw008Ad | 3-Jan-16 | Half Moon Island, SouthShetland | *Pygoscelis antarcticus* | F: 5'-TTATATCATCTGTTTTCTTTTCGTATGCTAACTCT-3'  R: 5'-CAGCAAAACTCATAGAATACGTAGATGATAACATA-3' |
| MT196265 | AntCM4 | I | CPHALFsw009Ad | 3-Jan-16 | Half Moon Island, SouthShetland | *Pygoscelis antarcticus* | F: 5'-TTATATCATCTGTTTTCTTTTCGTATGCTAACTCT-3'  R: 5'-CAGCAAAACTCATAGAATACGTAGATGATAACATA-3' |
| MT196266 | AntCM4 | I | CPHALFsw010Ad | 3-Jan-16 | Half Moon Island, SouthShetland | *Pygoscelis antarcticus* | F: 5'-TTATATCATCTGTTTTCTTTTCGTATGCTAACTCT-3'  R: 5'-CAGCAAAACTCATAGAATACGTAGATGATAACATA-3' |
| MT196230 | AntCM5 | I | CPBAILsw001Ad | 29-Dec-15 | Baily Head, Deception Island | *Pygoscelis antarcticus* | F: 5'-GGGATTTACAGTTACGAATATATGGCTGTATTTTA-3'  R: 5'-CCTCCCTCATTGCTACTAGATGATTTTATAAAAAT-3' |
| MT196231 | AntCM5 | I | CPBAILsw002Ad | 29-Dec-15 | Baily Head, Deception Island | *Pygoscelis antarcticus* | F: 5'-GGGATTTACAGTTACGAATATATGGCTGTATTTTA-3'  R: 5'-CCTCCCTCATTGCTACTAGATGATTTTATAAAAAT-3' |
| MT196232 | AntCM5 | I | CPBAILsw003Ad | 29-Dec-15 | Baily Head, Deception Island | *Pygoscelis antarcticus* | F: 5'-GGGATTTACAGTTACGAATATATGGCTGTATTTTA-3'  R: 5'-CCTCCCTCATTGCTACTAGATGATTTTATAAAAAT-3' |
| MT196233 | AntCM5 | I | CPBAILsw005Ad | 29-Dec-15 | Baily Head, Deception Island | *Pygoscelis antarcticus* | F: 5'-GGGATTTACAGTTACGAATATATGGCTGTATTTTA-3'  R: 5'-CCTCCCTCATTGCTACTAGATGATTTTATAAAAAT-3' |
| MT196254 | AntCM5 | I | CPHALFsw001Ad | 3-Jan-16 | Half Moon Island, SouthShetland | *Pygoscelis antarcticus* | F: 5'-GGGATTTACAGTTACGAATATATGGGTGTATTTTA-3'  R: 5'-CCTCCCTCATTGCTACTAGATGATTTTATAAAAAT-3' |
| MT196255 | AntCM5 | I | CPHALFsw002Ad | 3-Jan-16 | Half Moon Island, SouthShetland | *Pygoscelis antarcticus* | F: 5'-GGGATTTACAGTTACGAATATATGGGTGTATTTTA-3'  R: 5'-CCTCCCTCATTGCTACTAGATGATTTTATAAAAAT-3' |
| MT196256 | AntCM5 | I | CPHALFsw003Ad | 3-Jan-16 | Half Moon Island, SouthShetland | *Pygoscelis antarcticus* | F: 5'-GGGATTTACAGTTACGAATATATGGGTGTATTTTA-3'  R: 5'-CCTCCCTCATTGCTACTAGATGATTTTATAAAAAT-3' |
| MT196257 | AntCM5 | I | CPHALFsw005Ad | 3-Jan-16 | Half Moon Island, SouthShetland | *Pygoscelis antarcticus* | F: 5'-GGGATTTACAGTTACGAATATATGGGTGTATTTTA-3'  R: 5'-CCTCCCTCATTGCTACTAGATGATTTTATAAAAAT-3' |
| MT196258 | AntCM5 | I | CPHALFsw008Ad | 3-Jan-16 | Half Moon Island, SouthShetland | *Pygoscelis antarcticus* | F: 5'-GGGATTTACAGTTACGAATATATGGGTGTATTTTA-3'  R: 5'-CCTCCCTCATTGCTACTAGATGATTTTATAAAAAT-3' |
| MT196259 | AntCM5 | I | CPHALFsw009Ad | 3-Jan-16 | Half Moon Island, SouthShetland | *Pygoscelis antarcticus* | F: 5'-GGGATTTACAGTTACGAATATATGGGTGTATTTTA-3'  R: 5'-CCTCCCTCATTGCTACTAGATGATTTTATAAAAAT-3' |
| MT196260 | AntCM5 | I | CPHALFsw010Ad | 3-Jan-16 | Half Moon Island, SouthShetland | *Pygoscelis antarcticus* | F: 5'-GGGATTTACAGTTACGAATATATGGGTGTATTTTA-3'  R: 5'-CCTCCCTCATTGCTACTAGATGATTTTATAAAAAT-3' |
| MT196235 | AntCM6 | I | CPBAILsw001Ad | 29-Dec-15 | Baily Head, Deception Island | *Pygoscelis antarcticus* | F: 5'-ATGTATTGTAGGTGTCATAGAAGGAAAGTAGTATC-3'  R: 5'-CATCAACTACTGATATTGTTTGTACAAAAGTAACA-3' |
| MT196236 | AntCM6 | I | CPBAILsw001Ch | 29-Dec-15 | Baily Head, Deception Island | *Pygoscelis antarcticus* | F: 5'-ATGTATTGTAGGTGTCATAGAAGGAAAGTAGTATC-3'  R: 5'-CATCAACTACTGATATTGTTTGTACAAAAGTAACA-3' |
| MT196237 | AntCM6 | I | CPBAILsw002Ad | 29-Dec-15 | Baily Head, Deception Island | *Pygoscelis antarcticus* | F: 5'-ATGTATTGTAGGTGTCATAGAAGGAAAGTAGTATC-3'  R: 5'-CATCAACTACTGATATTGTTTGTACAAAAGTAACA-3' |
| MT196238 | AntCM6 | I | CPBAILsw002Ch | 29-Dec-15 | Baily Head, Deception Island | *Pygoscelis antarcticus* | F: 5'-ATGTATTGTAGGTGTCATAGAAGGAAAGTAGTATC-3'  R: 5'-CATCAACTACTGATATTGTTTGTACAAAAGTAACA-3' |
| MT196239 | AntCM6 | I | CPBAILsw003Ad | 29-Dec-15 | Baily Head, Deception Island | *Pygoscelis antarcticus* | F: 5'-ATGTATTGTAGGTGTCATAGAAGGAAAGTAGTATC-3'  R: 5'-CATCAACTACTGATATTGTTTGTACAAAAGTAACA-3' |
| MT196240 | AntCM6 | I | CPBAILsw003Ch | 29-Dec-15 | Baily Head, Deception Island | *Pygoscelis antarcticus* | F: 5'-ATGTATTGTAGGTGTCATAGAAGGAAAGTAGTATC-3'  R: 5'-CATCAACTACTGATATTGTTTGTACAAAAGTAACA-3' |
| MT196241 | AntCM6 | I | CPBAILsw004Ad | 29-Dec-15 | Baily Head, Deception Island | *Pygoscelis antarcticus* | F: 5'-ATGTATTGTAGGTGTCATAGAAGGAAAGTAGTATC-3'  R: 5'-CATCAACTACTGATATTGTTTGTACAAAAGTAACA-3' |
| MT196242 | AntCM6 | I | CPBAILsw004Ch | 29-Dec-15 | Baily Head, Deception Island | *Pygoscelis antarcticus* | F: 5'-ATGTATTGTAGGTGTCATAGAAGGAAAGTAGTATC-3'  R: 5'-CATCAACTACTGATATTGTTTGTACAAAAGTAACA-3' |
| MT196243 | AntCM6 | I | CPBAILsw005Ad | 29-Dec-15 | Baily Head, Deception Island | *Pygoscelis antarcticus* | F: 5'-ATGTATTGTAGGTGTCATAGAAGGAAAGTAGTATC-3'  R: 5'-CATCAACTACTGATATTGTTTGTACAAAAGTAACA-3' |
| MT196244 | AntCM6 | I | CPBAILsw005Ch | 29-Dec-15 | Baily Head, Deception Island | *Pygoscelis antarcticus* | F: 5'-ATGTATTGTAGGTGTCATAGAAGGAAAGTAGTATC-3'  R: 5'-CATCAACTACTGATATTGTTTGTACAAAAGTAACA-3' |
| MT196280 | AntCM7 | I | CPHALFsw003Ad | 3-Jan-16 | Half Moon Island, SouthShetland | *Pygoscelis antarcticus* | F: 5'-CAAAACAGAAAATAATATAACAGATCTAGACGCAG-3'  R: 5'-TAAACTTCAATAATATCTACATGCATTGTTGTTGC-3' |
| MT196281 | AntCM7 | I | CPHALFsw004Ad | 3-Jan-16 | Half Moon Island, SouthShetland | *Pygoscelis antarcticus* | F: 5'-CAAAACAGAAAATAATATAACAGATCTAGACGCAG-3'  R: 5'-TAAACTTCAATAATATCTACATGCATTGTTGTTGC-3' |
| MT196282 | AntCM7 | I | CPHALFsw010Ad | 3-Jan-16 | Half Moon Island, SouthShetland | *Pygoscelis antarcticus* | F: 5'-GAAGAATTTTACTTGACTTGAGGCATTTTCATATT-3'  R: 5'-GATTACCATCTAATTTTATACGCTTACAGCAATTA-3' |
| MT196234 | AntCM8 | I | CPBAILsw001Ad | 29-Dec-15 | Baily Head, Deception Island | *Pygoscelis antarcticus* | F: 5'-GTATTAATCCGACCTACAAAACGATTGTATATGAT-3'  R: 5'-CATCATAAGGATGTTTATAATCATCGACTAGCCAC-3' |
| MT196246 | AntCM8 | II | CPBOOTsw002Ad | 7-Jan-16 | Booth Island, West Antarctic peninsula | *Pygoscelis antarcticus* | F: 5'-TATAGCAGAAAGGCAAGAATAATGAATAATACAGC-3'  R: 5'-GCCTGGTATAACGCATTATATCATCAATAATCATA-3' |
| MT196245 | AntCM8 | II | CPBOOTsw002Ad | 7-Jan-16 | Booth Island, West Antarctic peninsula | *Pygoscelis antarcticus* | F: 5'-AGGAAGATAAAAATCCTACTGTAAAAGGTGAATTT-3'  R: 5'-TTTGAATATATTTGAGCACGTCATGTATGTTTCTA-3' |
| MT196288 | AntCM8 | II | GPBOOTsw003Ad | 17-Jan-16 | Booth Island, West Antarctic peninsula | *Pygoscelis papua* | F: 5'-TTTGAATATATTTGAGCACGTCATGTATGTTTCTA-3'  R: 5'-AGGAAGATAAAAATCCTACTGTAAAAGGTGAATTT-3' |
| MT196284 | AntCM9 | I | CPHALFsw001Ad | 3-Jan-16 | Half Moon Island, SouthShetland | *Pygoscelis antarcticus* | F: 5'-GTATTAGCAAATTAGCAGAATTAGCATACG-3'  R: 5'-GCTCTTGACTCATATTGGTTCTTATTTTTC-3' |
| MT196285 | AntCM9 | I | CPHALFsw002Ad | 3-Jan-16 | Half Moon Island, SouthShetland | *Pygoscelis antarcticus* | F: 5'-GTATTAGCAAATTAGCAGAATTAGCATACG-3'  R: 5'-GCTCTTGACTCATATTGGTTCTTATTTTTC-3' |
| MT196286 | AntCM10 | I | CPHALFsw008Ad | 3-Jan-16 | Half Moon Island, SouthShetland | *Pygoscelis antarcticus* | F: 5'-CGATAATAACACTGCGGTTCTAGATATACT-3'  R: 5'-CTAAATATTTTTAGCGATACACTTTTGGCG-3' |
| MT196287 | AntCM10 | I | CPHALFsw010Ad | 3-Jan-16 | Half Moon Island, SouthShetland | *Pygoscelis antarcticus* | F: 5'-CGATAATAACACTGCGGTTCTAGATATACT-3'  R: 5'-CTAAATATTTTTAGCGATACACTTTTGGCG-3' |
| MT196283 | AntCM11 | I | CPHALFsw005Ad | 3-Jan-16 | Half Moon Island, SouthShetland | *Pygoscelis antarcticus* | F: 5'-GGAGATTTGTTTGTAGTTGGGATGAGGTATTAAAG-3'  R: 5'-GGTAAAATCATTAATTGCATGTAAATCTTAGGTAATCTTACCC-3' |
| MT196267 | AntCM12 | I | CPHALFsw003Ad | 3-Jan-16 | Half Moon Island, SouthShetland | *Pygoscelis antarcticus* | F: 5'-CTTTATTAGCATACTGGTCTTAATACAACACTTCT-3'  R: 5'-CCATAAGTGAAAAATTAAGTAAGACACCATAACAA-3' |
| MT196268 | AntCM12 | I | CPHALFsw004Ad | 3-Jan-16 | Half Moon Island, SouthShetland | *Pygoscelis antarcticus* | F: 5'-CTTTATTAGCATACTGGTCTTAATACAACACTTCT-3'  R: 5'-CCATAAGTGAAAAATTAAGTAAGACACCATAACAA-3' |
| MT196269 | AntCM12 | I | CPHALFsw008Ad | 3-Jan-16 | Half Moon Island, SouthShetland | *Pygoscelis antarcticus* | F: 5'-CTTTATTAGCATACTGGTCTTAATACAACACTTCT-3'  R: 5'-CCATAAGTGAAAAATTAAGTAAGACACCATAACAA-3' |
| MT196270 | AntCM12 | I | CPHALFsw009Ad | 3-Jan-16 | Half Moon Island, SouthShetland | *Pygoscelis antarcticus* | F: 5'-CTTTATTAGCATACTGGTCTTAATACAACACTTCT-3'  R: 5'-CCATAAGTGAAAAATTAAGTAAGACACCATAACAA-3' |
| MT196271 | AntCM12 | I | CPHALFsw010Ad | 3-Jan-16 | Half Moon Island, SouthShetland | *Pygoscelis antarcticus* | F: 5'-CTTTATTAGCATACTGGTCTTAATACAACACTTCT-3'  R: 5'-CCATAAGTGAAAAATTAAGTAAGACACCATAACAA-3' |
